# Support Needs and Parent Outcomes in Arab Families of Children with Autism Living in the United Kingdom

**DOI:** 10.3390/brainsci12081114

**Published:** 2022-08-22

**Authors:** Barah Alallawi, Richard Hastings, Nura Aabe

**Affiliations:** 1 Centre for Educational Development Appraisal and Research, University of Warwick, Coventry CV4 8UW, UK; 2 Autism Independence, Silai Centre, Bristol BS5 0ES, UK

**Keywords:** autism, support needs, Arab family, parent outcomes, survey

## Abstract

Research on the experiences of Arab families of children with autism living in non-Arab countries is scarce. A survey investigated the support needs, psychological distress, and parental relationships of Arab parents (n = 100) of children with autism living in the United Kingdom (UK). The survey consisted of five main questionnaires: a demographic questionnaire, the GO4KIDDS Brief Adaptive Scale, the Family Needs Survey, the Strengths and Difficulties Questionnaire, and the Hospital Anxiety and Depression Scale. Descriptive analysis indicated that the most frequently identified support needs were information, community services, and explaining to others. Parents reported high levels of psychological distress, a high level of parental relationship satisfaction with their spouse, and few parental disagreements about their child with autism. Regression analyses showed that increased child behavior problems predicted more total family needs. Higher levels of child prosocial behavior, the better health status of parents, and a larger number of children in the family were associated with lower levels of parental psychological distress. A longer time living in the UK was associated with more parental disagreement over issues related to the child with ASD. Reducing child behavior problems and increasing child prosocial behavior may be important targets for support and intervention to improve outcomes for Arab parents of autistic children.

## 1. Introduction

The impact of autism is multidimensional as it affects the family financially, socially, and in terms of employment. Family members’ outcomes may also be affected when raising a child with autism. For example, parents of children with autism report having elevated parenting stress [1,2,3] and psychological distress, including depression and anxiety symptoms [4,5,6], compared to parents of children who do not have autism. There is also evidence that parenting stress persists throughout the course of a child’s early development [7,8] and continues into adolescence and adulthood [9].

Raising a child with autism may also affect family systems and subsystems. For example, the everyday life and long-term outlook of couples raising children may be affected. Due to the additional practical, emotional and financial challenges of raising a child with autism, parents may have less time to spend with one another, struggle to manage their partner’s reaction to having a child with a disability, and encounter difficulties balancing their role as a partner and a parent [10]. Parents of children with autism have been found to report lower levels of relationship satisfaction when compared with parents who do not have children with disabilities [11,12,13,14] and parents who have children with intellectual disability [15] or Down syndrome [13]. In addition, Hartley et al. [16] reported that marital problems might persist over time in families raising children with autism.

Social support may help to alleviate the challenges associated with raising a child with autism. Research on parents of children with autism has found that social support is linked to lower levels of psychological distress [17], negative mood [18], depressive symptoms [19], fewer marital problems [20], as well as higher levels of parental relationship satisfaction [21]. Dunst, Trivette and Hamby [22] defined social support as the assistance an individual receives from others. Support can be emotional, psychological, physical, informational, instrumental, and material help that affects the receiver’s behavior either directly or indirectly [22]. Support can come from formal or informal sources [23]. An agency or organization provides formal support in the form of social, psychological, physical, or financial support. Informal support is the assistance provided by someone in the individual’s network that involves family, friends, neighbors, and parents of other children with disabilities [24]. 

Albanese, Miguel and Koegel [25] stressed that professionals should start with an assessment of the needs and wishes of the family to initiate appropriate social support. Dunst, Trivette, and Jenkins [26] (p. 13) define need as: “an individual’s judgment of the discrepancy between actual states or conditions and what is normative, desired, or valued from a help seeker’s and not a help giver’s perspective.” A comprehensive assessment of family needs can enhance the formulation of meaningful interventions to address parents’ particular concerns and desires [27,28]. 

Bailey and Simeonsson [29] designed a measure, the Family Needs Survey (FNS), to assess the needs of 34 two-parent families participating in home-based intervention programs for infants with a variety of disabilities (average age = 14 months). The survey consisted of 35 items categorized into six derived clusters: needs for information, e.g., ‘I need more information about how to teach my child,’ needs for support, e.g., ‘I need to have more friends that I can talk to,’ explaining to others, e.g., I need help in explaining my child’s condition to other children,’ community services, e.g., ‘I need help locating a dentist who will see my child,’ financial needs, e.g., ‘I need more help in getting special equipment form my child’s needs,’ and family functioning, e.g., ‘our family needs help deciding on and doing recreational activities.’ The FNS was used by Ellis et al. [30] to assess the needs of 91 families of children with developmental disabilities (90% with a diagnosis of autism) up to the age of 22 years. Results showed that parents’ greatest reported needs were for information, support, and community services. Financial assistance was the lowest reported need. In addition, Ellis et al. [30] evaluated situational variables that predicted needs and found that parents of younger children with autism reported the greatest needs overall. In contrast, parents’ age, education, income, number of siblings, and participation in support services did not predict self-reported needs. Fewer reported needs were identified by families who had a child enrolled in a residential setting. More recently, Hodgetts, Zwaigenbaum, and Nicholas [31] assessed the needs of 143 families of children with autism (2–18 years). The study found that the most commonly unmet needs were the need for information about services for the child both now and in the future, family support, and respite care. The quality of professional support available was perceived positively. The child’s age, mother’s age, and household income were significant predictors of more total needs. Having an older child or mother, lower income, and the child’s disruptive behaviors predicted more total unmet needs. Children’s language or intellectual abilities did not predict needs. 

Different ethnic and cultural groups can vary considerably in their beliefs about disability, the nature of family and community supports, ethnomedical practices, and the utilization of professional services [32,33]. For example, in comparing Chinese and Malay Muslim mothers of children with intellectual disabilities, Ow, Tan, and Goh [34] found that patterns of reported social support differed by culture. The Malay Muslim mothers did not have formal support sources, while approximately half of the Chinese mothers did. Ow et al. [34] argued that religious and cultural beliefs, cognitive frameworks, and satisfaction with informal supports might influence different populations’ formal support needs and service use. Language barriers may also impede access to social support [35,36]. Bailey et al. [37] argued that researchers had paid more attention to the differences between ethnic groups than across families within a given ethnic group, particularly when the ethnic group is a minority with respect to the majority culture and language. 

Following Bailey et al.’s [37] recommendation, findings from a recent systematic review [38] showed that research on the support needs of Arab families of children with autism is absent internationally. Further, there were only six studies on parent psychological distress or mental health in Arab families [39,40,41,42,43,44] and no studies about parental relationships in Arab families of children with autism. There were also only two studies focused on the experiences of Arab families of children with autism in Western countries: one qualitative study about Somali families of children with autism in the UK and their access to services [45] and a qualitative study about the stresses experienced by Somali families of children with autism in Canada [46]. To our knowledge, no published studies have focused on the support needs and parental outcomes in Arab families of children with autism living in the UK. Therefore, the main aims of the current exploratory study were to (1) describe support needs, psychological distress, and parental relationships of Arab families of children with autism living in the UK and (2) identify factors associated with these outcomes.

## 2. Material and Methods 

### 2.1. Participants

Participants were Arab primary caregivers (83 biological mothers and 17 biological fathers) of children with autism living in the UK. Ninety-three participants were married; the remainder were divorced, separated, or widowed. Mothers and fathers were well-educated: 22 had school leaving qualifications, 51 had a college or university degree, 15 held Master’s or Doctoral degrees, and only seven had no formal educational qualifications. Fifty of the parents were aged between 35 and 44 years old, 40 of them were between 25 and 34 years old, only one parent was under the age of 24, while nine of them were between the age of 45 and 54 (parents were asked to indicate age range not a specific age in years). Thirty-three parents were employed, and the remainder were unemployed. Seventy-two of the parents reported good health status, while 28 had fair or poor health status. As shown in Table 1, the participants were originally from 19 different Arab countries. The majority of them came from Iraq (12), Egypt (11), and Syria (11). They were all born in an Arab country other than the UK, and their time length in the UK ranged from one year to 38 years (M = 10.05, SD = 7.54). 

One family had three children under 18 years of age diagnosed with autism; three families had two children with autism, while the remainder reported having only one child with autism. On average, families had three children (M = 3.08, SD = 1.21; range 1–7) and two adults above 18 years of age currently living in the family home (M = 2.02, SD = 0.49; range 1–5). A family socioeconomic disadvantage variable was created, including three indicators summed as a total score (0–3): the parent had no educational qualifications (scoring one), reporting of family financial hardship [rating of ‘finding it quite difficult’ or ‘finding it very difficult’ in response to ‘how well would you say you and your husband/wife/partner are managing financially these days?’] (scoring one), and the household income below £600 per week (scoring one). Only one family had no disadvantage indicators, 19 families had one, 70 families had two, and 10 families had three disadvantage indicators. The mean score for this disadvantage index was 1.89 (SD = 0.57). 

The children with autism were between the ages of 4 and 15 years of age (M = 100.02, SD = 35.297) and were predominantly male (78%). According to parental reports, 99 of the children had received a diagnosis of autism and one of Asperger Syndrome. Thirty-three families reported that it took more than one year from raising their initial concerns about their child to obtaining the diagnosis. In contrast, other families reported that it took about one year (30 families), about six months (24 families), and about three months (13 families).

### 2.2. Measures

Participants completed an anonymous online or postal survey that included: a demographic questionnaire designed for the present research to assess characteristics described above and questionnaires measuring child behavior problems, family needs, and parental psychological distress.

Child measures

The Strengths and Difficulties Questionnaire (SDQ) [47] was used to measure the behavioral and emotional problems of a child with autism. This measure comprises 25 items assessing five domains: prosocial behavior, e.g., considerate of other people’s feelings; and four problem behavior domains including emotional problems, e.g., often complains of headaches, stomach aches or sickness, conduct problems, e.g., often fights with other children, hyperactivity, e.g., constantly fidgeting or squirming, and peer relationship problems, e.g., rather solitary, tends to play alone. Respondents rated statements about their child as either not true, somewhat true, or certainly true. The SDQ total difficulties score was generated by summing emotional problems, conduct problems, hyperactivity, and peer relationship problems as the measure of children’s behavioral and emotional problems. A higher score is indicative of greater behavioral and emotional difficulties. The SDQ has demonstrated good levels of reliability and validity for use as a community screening tool for children and adolescents [48]. Research on children with autism suggested that the SDQ maintains good psychometric properties when used in this population [49,50]. In the present sample, a Cronbach’s alpha of 0.74 was obtained for the total difficulties score and 0.75 for the prosocial behavior subscale score. 

The GO4KIDS Brief Adaptive scale was used to measure adaptive behavior [51]. This measure contains eight items that assess a child’s adaptive behavior across four domains: support needs, communication, socialization, and self-help skills. A new item designed to cover additional communication skills ‘How much does your child use alternative methods of communication to communicate? (e.g., signing, symbol systems, Picture Exchange Communication System) (If applicable)’ was added to the measure. Each item is rated on a five-point scale, with higher scores indicating greater skill level and greater independence. An overall adaptive behavior score was derived by summing the ratings on the nine items [51]. Satisfactory reliability and validity have been demonstrated by parents of children and youth with developmental disabilities [51]. Cronbach’s alpha for the total score in the current study was 0.81.

Parental measures

Parents’ psychological distress was measured using the Hospital Anxiety and Depression scale (HADS) [52]. Although initially developed for residential psychiatric populations, the HADS has been used widely in community research. This measure comprises 14 items, with seven assessing depression, e.g., ‘I feel as if I am slowed down,’ and seven assessing anxiety, ‘e.g., ‘I feel tense or wound up.’ A dimensional approach was taken for the analyses in the present study, with a total score for all 14 items being used as an index of psychological distress. Previous research with parents of children with developmental disabilities has shown that the HADS maintains good reliability within these populations [53,54,55]. For the current sample, Cronbach’s alpha level for the psychological distress total score was 0.82. 

The Family Needs Survey (FNS) [56] was used to assess family needs. This survey consists of 35 items reflecting needs commonly expressed by parents of children with disabilities. The items are organized into seven domains: information, e.g., ‘how to teach my child,’ family and social support, e.g., ‘talking with someone in my family about concerns,’ financial assistance, e.g., ‘getting any special equipment my child needs,’ explaining to others, e.g., ‘explaining my child’s condition to his or her siblings,’ child care, e.g., ‘locating babysitters for my child,’ professional support, e.g., ‘meeting with a counsellor,’ and community services, e.g., ‘locating a doctor who understands me and my child needs.’ In the present study, parents rated on a 3-point scale: 1 indicated a response of ‘no support needed’, 2 indicated ‘a little support needed’ and 3 indicated ‘a lot of support needed’. Previous research with parents of children with developmental disabilities has reported that the FNS maintains good reliability within these populations [30,37]. Cronbach’s alpha for FNS total score in the present study was 0.88. 

Parental relationship satisfaction was measured using a scale that described the degree of happiness with a spouse or partner. Parents selected options from 1–7, where ‘1’ represented a very unhappy relationship and ‘7’ was a very happy relationship. This outcome was dichotomized into two categories: high relationship satisfaction and lower relationship satisfaction. This was because after running descriptive statistics, we found that the majority of responses in the sample fell into ‘6 and 7’ options, while a few responses fell into ‘1–5’ options. Thus, scores of 6 or 7 were classified as high relationship satisfaction, while the remainder of scores were classified as lower relationship satisfaction. Parental disagreement over issues related to their child with ASD was rated on a 6-point scale from ‘never’ to ‘more than once a day.’ These two single-item measures were the same as those used in a recent large-scale UK family survey [57].

### 2.3. Procedure

Translation process

Three questionnaires (demographic questionnaire and the parent relationship items, the GO4KIDS Brief Adaptive scale, and the FNS) were translated into Arabic in addition to participant information sheets and consent forms. There were already Arabic versions of the SDQ [58] and the HADS [59]. Therefore, depending on respondents’ preferences, the survey was available for completion in English or Arabic. We used the following translation procedures.

Step 1: Forward translation

After obtaining the authors’ permission to use the GO4KIDS Brief Adaptive scale and translate it into Arabic, the first researcher translated the demographic questionnaire, the GO4KIDS Brief Adaptive scale, and the FNS. A literal translation was avoided. Rather, the meaning of the statements as a whole unit was considered. Two additional bilingual individuals were also asked to translate the questionnaires into Arabic. A comparison was made between the first researcher’s translation and the other two individuals’ translations. An initial Arabic version was produced after a few alterations were made at this stage. Three bilingual individuals who are experienced in working with children with disabilities were then asked to check the suitability of the translation. The final Arabic versions were produced after obtaining a few comments and feedback from these bilingual individuals. A final Arabic version was approved, in terms of the level of written standard Arabic, by an expert in the Arabic language.

Step 2: Back translation

The Arabic translation resulting from the end of the first step was given to another bilingual individual who had a Ph.D. in psychology from the UK and was experienced in translation. She was asked to back-translate the three new Arabic questionnaires into English.

Step 3: Comparison and revision

The original English questionnaires and the back-translated English versions, which resulted from Step 2, were compared by an English speaker to check for mismatches. Some items in the back translation had a meaning that was too far from the original English. A few alterations in Arabic were made in the Arabic version to help convey a meaning closer to the original English versions.

Recruitment of participants

Recruitment was initiated upon receiving approval from the Humanities and Social Sciences Research Ethics Committee at the {removed for blind review}. Primary caregivers of children with autism were are aged between 4 years and 15 years 11 months, from Arab families living in the UK, were eligible to participate in the study if they self-identified as originating from any one of the following 22 Arab League states: Algeria, Somalia, Egypt, Libya, Sudan, Tunisia, Morocco, Mauritania, Djibouti, Bahrain, United Arab Emirates, Oman, Kuwait, Qatar, Saudi Arabia, Yemen, Jordan, Syria, Iraq, State of Palestine, Lebanon, or Comoros. Primary parental caregivers were not necessarily the child’s mother but the adult who most of the time cared for the child with autism. Parents may also have been biological, adoptive, or foster parents. 

A variety of different routes were used to contact Arab primary caregivers of children with autism in the UK, such as via autism and child disability charities or special schools that provide services to children with autism, and online via Facebook, Twitter, and WhatsApp groups, recruitment flyers in both English and Arabic, and presentations at meetings of parent groups. Recruitment information included a brief description of the study and links to access the survey in both English and Arabic. A reminder letter was emailed to all previous contacts one month after sending a recruitment email. Another reminder letter was also emailed before closing the survey. Families received no payment or other benefit for cooperating with the study. Participant information sheets and consent statements are accompanied by both online and postal surveys.

During the recruitment process, Somali language versions of the information sheet and consent forms were also developed to enable responses of Somali families. Somali parents attending a support group were offered an option to complete the survey face-to-face. Nine parents participated in the survey by completing it face-to-face. 

### 2.4. Data Analysis 

Before the main statistical analyses were conducted, the main study variables (family needs and levels of parental psychological distress) were tested for the normality of their distributions using skewness and kurtosis tests. The values of skewness and kurtosis tests revealed that the variables approximated a normal distribution. Data analysis proceeded in several steps. First, descriptive analysis was conducted to identify family needs, parental psychological distress levels, relationship satisfaction levels, and parental disagreement about their child with autism. Second, the analyses used linear regression models. Three analyses were conducted, one predicting family needs, the second predicting psychological distress levels in parents, and the third predicting parental disagreement. Child predictor variables included the SDQ prosocial scale, the total score of the GO4KIDDS Brief Adaptive scale, the total difficulties score of the SDQ, child’s age, and gender; and parent predictor variables included current health status, employment status, length of time living in the UK, socioeconomic status [using the disadvantage index score], number of children in the family, and number of children with autism in the family. All these variables were entered into the regression models simultaneously, and the residuals were inspected. Third, logistic regression was conducted to examine the child and parent predictor variables for relationship satisfaction (score dichotomously: high vs. lower parental relationship satisfaction-see Measures). There were no missing data, except that participants’ responses of ‘can’t say’ to the parent relationship measures were considered missing. Statistical analyses in the study were conducted using IBM SPSS Statistics 25.0^®^.

## 3. Results

### 3.1. Descriptive Analysis

As shown in Table 2, the mean score for the overall level (total score) of reported family needs was 73.30 (SD = 11.27). Subscales or topic areas with the highest means included need for information (M = 2.73; SD = 0.421), community services (M = 2.62; SD = 0.608) and explaining to others (M = 2.07; SD = 0.576). The most commonly reported needs were the need for information about currently available services as well as services that the child might receive in the future; information regarding how to teach and handle the child’s behavior; meeting and talking with other parents who have a child with autism; locating a dentist for the child; and finding reading material about other families who have a child with autism. The least frequently reported needs were meeting with an imam, priest, or rabbi; the need for help in deciding who will do household chores, childcare, and other family tasks; needs related to getting appropriate care for the child in a mosque, church or synagogue during religious services; the need for counseling or help in getting a job; and deciding on and doing family recreational activities. 

On average, parents had a total score on the HADS of 17.65 (SD = 6.30). For the anxiety subscale, the mean score was 9.14 (SD = 4.02); for the depression subscale, it was 8.51 (SD = 3.21). A cut-off score of 11 [60] was used as an indication of the presence of either depression or anxiety (participants scoring 11 and above). Using this cut-off, 27% of parents were identified as likely to have depression, and 37% were identified as likely to have anxiety.

Sixty-two percent of the parents reported high relationship satisfaction with their spouse, while 23% reported lower relationship satisfaction. Descriptive statistics on parental disagreement revealed that 44% of the parents reported no disagreement over issues related to the child, 17% reported disagreements ‘less than once a week’, once a week by 11%, and several times a week by 6%. In comparison, disagreements once a week and more than once a week were reported by 1% and 4%, respectively. 

### 3.2. Regression Analyses 

For total family needs, the regression model explained 14% of the variance (Adjusted R2 = 0.141). Only the total difficulties score of the SDQ made a significant contribution to the prediction of total family needs (β = 0.326), with more child behavior problems associated with increased family needs. No other predictors were statistically significant. 

For psychological distress levels, multiple regression analysis showed that the child’s prosocial behavior scale, the health status of parents, and the number of children in the family were significant predictors (β = −0.423, −0.259, and −0.218), respectively. Having a child with lower levels of prosocial behavior, the parent having poor health, and fewer children in the family were all associated with increased psychological distress (Table 3). No other predictors were statistically significant. The model explained 23% of the variance in total psychological distress (Adjusted R2 = 0.228). As shown in Table 3, length of time living in the UK was a significant predictor (β = 0.296) of parental disagreement about the child with autism (living longer in the UK was associated with increased parental disagreement). No other predictors were statistically significant. This regression model explained 20% of the variance in the parental disagreement outcome (Adjusted R2 = 0.202).

Logistic regression analysis revealed that the model as a whole explained between 14.5% (Cox and Snell R2) and 21% (Nagelkerke R2) of the variance in parental relationship satisfaction and correctly classified 72.9% of cases. As shown in Table 4, only parents’ employment status predicted parental relationship satisfaction. Employed parents were less likely to report a high relationship satisfaction with their spouse or partner by a factor of 0.189 (95% CI: 0.046, 0.777). 

## 4. Discussion

Raising a child with autism likely generates needs for families across many domains of life. The pattern of needs reported by the parents in the current study is consistent with that found in previous research (e.g., [30,31,37,61]). For example, at the domain level, information needs have consistently been higher than other needs domains. In addition, at the item level, needs related to the child’s condition (e.g., need for information about services for the child both now and in the future, information on how to teach and handle the child’s behavior, meeting with other parents who have a child with autism, locating a dentist for the child and finding reading material about other families who have a child with autism) were generally rated higher than more general family needs such as meeting with an imam, the need for help in deciding who will do chores, counseling or help in getting a job, and deciding on and doing family recreational activities. 

Parents reported having high depression and anxiety levels, which are relatively consistent with the psychological distress profile of Arab families of children with autism in other research in Arab countries (e.g., [39,53]) but possibly higher than other UK parents of children with autism [54,62]. One might argue that the stigma of having a child with a disability is significantly more severe for parents in Arab cultures than in other cultures (e.g., Western European, Latin American, and South Asian). Thus, the perceived negative effect of having a child with a disability might be aggravated in Arab cultures and populations, leading to higher levels of psychological distress [42,63,64]. 

Most parents reported high levels of relationship satisfaction with their spouse or partner, which may be in contrast to other studies of parents raising a child with autism (e.g., [11,14,65]). Al-Kandari et al. [66] concluded that religion was the most frequently used coping strategy by Arab (Kuwaiti) mothers of children with autism. Religion can provide a supportive function among parents of children with autism. It may be positively correlated with increased parental relationship satisfaction and inversely correlated with relationship disagreement in parents of children with autism [67,68]. This might also explain the finding that most parents in the current sample reported no disagreement with their spouse over issues related to the child with autism. 

Consistent with previous research (e.g., [69,70]), the level of the child with autism’s behavior problems was a significant predictor of total reported family needs. Previous research also found that the most consistent and robust predictor of parental psychological distress levels, including depression and anxiety, was the child’s behavior problems [1,42]. In the present study, we found that the child’s prosocial behavior (not behavior problems) was a significant predictor of parental psychological distress levels. In advance of replication, it is unclear whether this may be a cultural difference or simply a feature of the current sample of families. Associations between parental distress and parents’ health status and the number of children in the family are consistent with previous research (e.g., [71,72]). The finding that employment status was associated with lower parental relationship satisfaction may be a function of primary parental caregivers (mainly mothers) having long, exhausting, tightly scheduled days working outside the home and at the same time caring for their child with autism. Employed parents may also have less time to spend with one another, having more day-to-day demands imposed on them when balancing their different roles as a partner, a parent, and an employee. We also found that the longer families had been in the UK, the more parental disagreement over issues related to the child with autism was reported, although it is unclear why this might be the case. 

The study has provided an initial insight into support needs, psychological distress, and parental relationship of Arab families of children with autism living in the UK and unique quantitative data on Arab families of children with autism living outside of Arab countries [37]. However, it is important to note that data were predominantly obtained from mothers. Therefore, future research intentionally sourcing data from the perspective of Arab fathers of children with autism is needed. Understanding and identifying the needs of Arab families of children with autism living in the UK may inform support and services needed. For example, based on our findings, parents reported the need for information on how to teach their children with autism. Additional research on Arab parent-mediated educational interventions for their children with autism is needed. In addition, support groups could be provided to the families to address their need to acquire information about current and future services for their children with autism. Although significant amounts of the variance in each main study outcome were explained in the regression models, it is important to note that a large amount of the variance was not explained by factors in the analyses. Additional predictor variables at the child, family, or contextual (e.g., cultural) levels should be examined in future research.

There is a further limitation that should be acknowledged when interpreting and considering the generalizability of the results of this study. It is unclear whether the families in our sample are representative of the population of Arab families that have a child with autism living in the UK. In particular, the sample is relatively small, and the response rate was essentially unknown as parents were recruited through various advertisements. This study needs to be replicated with larger samples. 

## 5. Conclusions 

This study has provided information on support needs, psychological distress, and parental relationships of Arab parents of children with autism living in the UK. This information will help better understand those parents and find ways to support them effectively, which can benefit their children with autism and their family as a whole. The findings from this study could inform professionals, funders, service providers, and policymakers in tailoring services to best meet Arab family needs. 

## Figures and Tables

**Table 1:** Participants’ Arab identity.

Country	Number of Participants	Country	Number of Participants	Country	Number of Participants
Egypt	11	Lebanon	3	Somalia	9
Sudan	5	Kuwait	2	Jordan	6
Morocco	6	Qatar	1	Palestine	6
Yemen	4	Algeria	6	Bahrain	2
Tunisia	5	Iraq	12	Oman	2
United Arab Emirates	2	Saudi Arabia	2		
Libya	5	Syria	11		

**Table 2:** Total and subscales scores of the Family Needs Survey.

Subscale	Mean	SD
Information	19.08	2.946
Family and social support	13.74	3.749
Financial assistance	11.44	3.639
Explaining to Others	10.35	2.879
Child Care	5.18	1.702
Professional Support	5.64	1.487
Community Services	7.87	1.824
Total	73.3	11.266

**Table 3:** Results of linear regression analysis of family needs, psychological distress, and parental disagreement.

Predictor Variables	Family Needs			Psychological Distress			Parental Disagreement		
**Child variables**	B	β	*p*	B	β	*p*	B	β	*p*
Age	−0.036	−0.113	0.350	−0.011	−0.095	0.406	−0.009	−0.217	0.088
Gender	0.170	0.006	0.953	−0.845	−0.083	0.412	−0.347	−0.108	0.362
Prosocial total score	−0.459	−0.104	0.441	−0.698	−0.423	0.001	−0.021	−0.041	0.778
GO4KIDS total score	−0.320	−0.176	0.203	0.043	0.063	0.632	0.043	0.193	0.189
SDQ total problems	0.748	0.326	0.009	0.086	0.100	0.390	−0.021	−0.041	0.778
**Parent variables**									
Health status	−0.896	−0.036	0.769	−2.421	−0.259	0.028	−0.228	−0.076	0.583
Employment status	0.759	0.032	0.782	0.142	0.016	0.884	0.030	0.011	0.932
Time length in the UK	−0.075	−0.050	0.651	0.031	0.056	0.593	0.056	0.296	0.013
Family disadvantage index	1.830	0.092	0.416	−0.425	−0.057	0.595	0.161	0.060	0.610
Number of children in the family	0.828	0.089	0.418	−0.758	−0.218	.039	−0.084	−0.072	0.538
Number of children with autism in the family	−2.426	−0.056	0.586	1.179	0.073	0.456	0.501	0.098	0.384

**Table 4:** Results of logistic regression analysis predicting parental relationship satisfaction.

Predictor Variables	B	S.E.	Wald	*p*	Odds Ratio	95% C.I. for Odds Ratio	
**Child variables**						Lower	Upper
Age	0.006	0.009	0.353	0.552	1.006	0.987	1.025
Gender	0.29	0.732	0.157	0.692	1.337	0.318	5.617
Prosocial total score	0.159	0.147	1.173	0.279	1.172	0.879	1.562
GO4KIDS total score	−0.018	0.065	0.079	0.779	0.982	0.865	1.114
SDQ total score	−0.066	0.068	0.941	0.332	0.936	0.820	1.070
**Parent variables**							
Health status	1.306	0.783	2.784	0.095	3.692	0.796	17.120
Employment status	−1.664	0.72	5.335	0.021	0.189	0.046	0.777
Time length in the UK	−0.029	0.038	0.574	0.449	0.971	0.901	1.047
Family disadvantage	0.985	0.619	2.536	0.111	2.679	0.797	9.009
Number of children in the family	0.114	0.243	0.221	0.639	1.121	0.696	1.805
Number of children with autism in the family	−1.674	1.669	1.005	0.316	0.188	0.007	4.945
Constant	1.033	2.555	0.163	0.686	2.809		

## Data Availability

Not applicable.
